# #GenderAffirmingHormoneTherapy and Health Information on TikTok: Thematic Content Analysis

**DOI:** 10.2196/66845

**Published:** 2025-04-29

**Authors:** Julia Rose Beatini, Nora Yanyi Sun, Julianna K Coleman, Maetal E Haas-Kogan, Andrea Pelletier, Deborah Bartz, Alex Sogomon Keuroghlian

**Affiliations:** 1 Thomas Jefferson University, Philadelphia, PA, United States; 2 Harvard University, Cambridge, MA, United States; 3 Harvard Medical School, Boston, MA, United States; 4 Department of Obstetrics and Gynecology, Brigham and Women’s Hospital, Boston, MA, United States; 5 Department of Psychiatry, Massachusetts General Hospital, 55 Fruit Street, Wang Building 8th Floor, Boston, MA, 02114, United States, 1 (857) 313-6688, 1 (617) 267-0764; 6 The National LGBTQIA+ Health Education Center, The Fenway Institute, Boston, MA, United States

**Keywords:** transgender, gender diverse, transgender and gender diverse, TGD, gender fluid, online platform, social media, gender affirming, hormone therapy, gender-affirming hormone therapy, GAHT, social media content, media information, social media analysis, TikTok, web scraper, hashtag, themes, qualitative content analysis, patient education materials assessment, PEMAT, Currency, Relevance, Authority, Accuracy, and Purpose, CRAAP, audiovisual materials, qualitative

## Abstract

**Background:**

Transgender and gender diverse people often turn to online platforms for information and support regarding gender-affirming hormone therapy (GAHT); however, analysis of this social media content remains scarce.

**Objective:**

We characterized GAHT-related videos on TikTok to highlight the implications relevant to GAHT prescribers.

**Methods:**

We used a web scraper to identify TikTok videos posted under the hashtags #genderaffirminghormonetherapy and #genderaffirminghormones as of November 2023. We identified recurrent themes via qualitative content analysis and assessed health education videos with the Patient Education Materials Assessment Tool for Audiovisual Materials (PEMAT-A/V) scale and a modified Currency, Relevance, Authority, Accuracy, and Purpose (CRAAP) test.

**Results:**

Out of 69 videos extracted, 71% (49/69) were created by GAHT users, 24.6% (17/69) were created by health care workers, and 21.7% (15/69) were created to provide health education. Themes included physical changes on testosterone, GAHT access, and combating misinformation and stigma surrounding GAHT. Health education videos scored highly on PEMAT-A/V items assessing understandability (mean 88.3%, SD 11.3%) and lower on actionability (mean 60.0%, SD 45.8%). On the CRAAP test, videos scored highly on the relevance, authority, and purpose domains but lower on the currency and accuracy domains.

**Conclusions:**

Discussions of GAHT on TikTok build community among transgender and gender diverse users, provide a platform for digital activism and resistance against legislation that limits GAHT access, and foster patient-provider dialogue. Educational videos are highly understandable and are created by reliable sources, but they vary in terms of currency and quality of supporting evidence, and they lack in actionability.

## Introduction

TikTok (ByteDance) is a short-form video-sharing application that boasts 97.6 million active users in the United States. Since TikTok’s spike in popularity in 2020, transgender and gender diverse (TGD) content creators have used the platform as a space to document and share their experiences with others. While TikTok has the potential to disseminate health information and improve access to gender-affirming care, it has come under scrutiny for spreading misinformation, bias, and hate speech [1].

Despite the lack of systematic analysis, the spread of information about gender-affirming care within the TGD TikTok community has been cited as an example of community-engaged knowledge exchange and peer-to-peer support [2]. Furthermore, TikTok has the potential to improve access to health information among communities that experience health inequities as the result of discrimination, because having positive impressions of knowledgeable professionals on social media may help decrease medical mistrust and enhance access to care offline [2]. Lowering medical mistrust among TGD communities is crucial, given that 24% of respondents in the 2022 US Transgender Survey reported avoiding medical care due to fear of mistreatment by a provider [3].

While TikTok videos have the potential to improve access to health information, peer support, and trust in medical professionals, TikTok may also be used to spread misinformation, disinformation, and hateful rhetoric against lesbian, gay, bisexual, transgender, queer, intersex, and all asexually and gender diverse (LGBTQIA+) people on the platform [4]. For example, the use of gender-affirming hormone therapy (GAHT), which involves administering hormones like estrogen and testosterone or puberty blockers to alter gendered physical characteristics among TGD youth, has increasingly been attacked; its controversy has led to online hate speech and, in several instances, threats of violence against hospitals and individual providers online [5,6].

Previous studies have queried TikTok to explore attitudes toward and experiences with other types of medical care, using qualitative methods to determine the content and tone of posts about medical interventions ranging from contraceptive methods to erectile dysfunction treatment [7-9]. Others have focused on analyzing the quality and accuracy of health information reported on the platform [10-15]. Their analyses yielded insights into misconceptions about care, the prevalence of inaccurate factual claims about treatment, and salient elements of individual experiences with care, all of which have the potential to inform how clinicians treat and counsel their patients. However, there have been no analyses of users’ attitudes towards, experience with, or knowledge about GAHT.

The aim of this study is to explore popular TikTok content posted under the hashtags #genderaffirminghormones and #genderaffirminghormonetherapy. Using previously validated methods, we (1) describe the demographic characteristics, attitudes, and affiliations of video content creators; (2) perform a qualitative analysis of video content to identify content themes; and (3) assess the understandability, actionability, and reliability of information presented in a subset of educational videos. In doing so, we aim to better understand the degree to which TikTok is a vehicle for sharing valuable information about GAHT and treatment access versus a potentiator of misinformation and harmful biases.

## Methods

### Data Extraction

We used the web-scraping application Apify (Apify Technologies s.r.o.) TikTok scraper to download all TikTok videos posted publicly under the hashtags #genderaffirminghormonetherapy and #genderaffirminghormones as of November 17, 2023; Apify provided all videos as MP4 files. While videos that fit the inclusion criteria may be available under alternate hashtags, only these two hashtags were selected for this study, as it was infeasible to scrape the vast content created under broader hashtags; additionally, users who are actively seeking information on GAHT would most likely search these two hashtags. The scrape included a total of 86 videos. We applied the following exclusion criteria: (1) non-English language video, (2) country codes in the European Union or China (based on differences in data usage agreements in these regions), (3) GAHT not mentioned in the video, (4) duplicate video, or (5) video posts removed following the scrape.

### Descriptive Analysis of the Content

For all eligible videos, we recorded the date posted and video duration, and TikTok engagement statistics, including the number of video views, likes, comments, shares, and number of creator fans. Through discussion and consensus, the first and second authors determined five main categories of videos after reviewing all videos (personal experience, health education, politics, creator opinion, and humor) and categorized each video. The sum, median, and IQR of engagement statistics were calculated for each type of video to best characterize the distribution of engagement; these metrics were selected given that specific videos may go viral on the platform, thereby skewing the data.

The first author then determined content creator demographics, including self-described gender identity and sexual orientation, for each video via the exploration of content creators’ public profiles, including the user, bios, current or previous videos, video captions, and comment responses [16]. Only explicitly stated identities from content creators were included to avoid assumptions about their identities; if no such statements were available, we marked the field as “unknown.” We selected “not applicable” if the account belonged to an organization rather than an individual. We similarly obtained creators’ GAHT user status, health care worker status, and organizational affiliations from information on their public profiles. Finally, the first and second authors individually rated each video on whether the creator displayed a positive (eg, supportive or encouraging), negative (eg, dismissive or transphobic), neutral (eg, purely informative), or ambiguous or mixed attitude (authors could not ascertain or agree on the creator’s intentions) toward GAHT. Through discussion, the authors came to a consensus on the final ratings of all videos.

### Quality Rating of the Content

We further analyzed a subset of health education videos to assess the understandability, actionability, and reliability of information presented. “Health education” videos contained at least one message about which creators aimed to inform viewers [17,18].

The first and second authors independently used the Patient Education Materials Assessment Tool for Audiovisual Materials (PEMAT-AV) to assess information understandability (ie, accessibility of the information presented for the layperson, including statements like “The material uses common, everyday language”; items 1‐13) and actionability (ie, the feasibility of implementing the information presented, including statements like “The material breaks down any action into manageable, explicit steps”; items 14‐17) of patient educational materials. Each item in the scale is rated as “agree” (1 point), “disagree” (0 points), or “not applicable.” The first and second author then compared each rating they assigned until they came to a consensus on all items. Finally, scores were calculated as a percentage of the possible points obtained for all items, excluding those rated as not applicable. Higher percentages suggest higher levels of understandability and actionability, with a threshold of >75% used to indicate “high quality” [19-21].

The senior author, who is a physician-scientist specialized in gender-affirming care, also used a modified Currency, Relevance, Authority, Accuracy, and Purpose (CRAAP) test to assess the reliability and accuracy of health information presented in educational videos [22]. The CRAAP test is used for the quantitative assessment of digital health information [23-25] by assessing five domains of information reliability: currency, relevance, authority, accuracy, and purpose. We adapted a previously published scale and scoring key by modifying the language and removing several items that were not applicable to audiovisual media [23]. Our adapted scale contained a total of 18 items (3 assessing currency, 4 assessing reliability, 3 assessing authority, 3 assessing accuracy, and 5 assessing purpose).

Overall scores ranged between 0 and 34, with higher scores suggesting higher reliability. Based on previous work using the CRAAP test, we considered a final score of <20% as unreliable, 20‐46% as reliable with caution, 46‐80% as good reliability, and >80% as excellent reliability [23]. We used Cronbach α to assess the interitem reliability of the scale (*α*=.88). The rubric for the modified CRAAP test is provided in Table 1.

**Table 1.** Modified Currency, Relevance, Authority, Accuracy, and Purpose (CRAAP)^a^ test.

Item	Scoring key			
	0	1	2	3
Currency (3 items, 5 points)				
Date created	>5 y	1‐5 y	<1 y	N/A^b^
Information outdated or debunked	Yes	No	N/A	N/A
Embedded links or suggested resources still accessible	None listed	No longer accessible	Still accessible	N/A
Relevance (4 items, 4 points)				
Information answers a central question	No	Yes	N/A	N/A
Information identifies an intended audience	No	Yes	N/A	N/A
Information appropriate for the needs of the intended audience	No	Yes	N/A	N/A
Information avoids overgeneralization	No	Yes	N/A	N/A
Authority (3 items, 5 points)				
Identity of the author or source	None identified	Medication user or patient	Expert in the field	N/A
Author’s credentials	None	Lived experience	Licensed medical professional	N/A
Author qualified to discuss the topic^c^	No	Yes	N/A	N/A
Accuracy (3 items, 7 points)				
Derivation of information	Unclear	Individual lived experience	Professional experience	Evidence-based review
Is the information supported by evidence^d^	No	Yes	N/A	N/A
What kind of evidence supports the claim	None	Individual lived experience	Expert/consensus opinion	Published evidence-based guidelines
Purpose (5 items, 8 points)				
Purpose of information	Advertisement	Persuading or entertaining	Informing	Teaching
Intentions or purpose clear	No	Yes	N/A	N/A
Nature of information	Propaganda	Opinion	Facts	N/A
Point of view appears objective and impartial	No	Yes	N/A	N/A
Political, ideological, cultural, or religious biases	Yes	No	N/A	N/A

a This modified CRAAP Test is developed from the original test by Sarah Blakeslee, which is licensed for adaptations under a Creative Commons Attribution 4.0 International License.

b N/A: not applicable

c Creators were considered qualified to discuss a topic if they either had confirmed credentials indicating relevant subject matter expertise and licensure relevant to the clinical topic, or lived experience in a patient testimonial.

d Information presented in videos was considered supported if contemporary scientific evidence was congruent with the information.

### Ethical Considerations

This study involves the analysis of publicly available data from TikTok; data from private accounts were not assessed as part of this project. Investigators only had access to creators’ account names while viewing videos during the initial scoring and coding of data. Any identifiable information, including account names, was removed from the data prior to dissemination. This study received a Not Human Subjects Research Determination from the Harvard Longwood Campus Institutional Review Board.

## Results

### Descriptive Analysis of the Content

Our search identified 84 videos posted between February 2022 (the earliest video searching the TikTok hashtag recalled) and October 2023; while data collection was performed in November 2023, no new videos under the hashtag were posted in November 2023. Fifteen videos were determined ineligible for analysis based on non-English language (n*=*6), country code in the European Union or China (n=1), GAHT not mentioned in the video (n=6), duplicate (n=1), or video post removed following the scrape (n=1). The remaining 69 videos were included in our analysis. The median (IQR) video duration was 56 (23‐73) seconds. Video characteristics and creator demographics are summarized in Table 2.

**Table 2.** Video characteristics and creator demographics of the 69 TikTok videos analyzed.

Video characteristics	n (%), N=69
Video type	
Personal experience	45 (65.2)
Health education	15 (21.7)
Politics	4 (5.8)
Creator opinion	4 (5.8)
Humor	1 (1.4)
Attitude toward GAHT^a^	
Positive	58 (84.1)
Negative	2 (2.9)
Neutral	5 (7.2)
Mixed or ambiguous	4 (5.8)
Medication referenced	
Testosterone	41 (59.4)
Estrogen	11 (15.9)
Antiandrogens	1 (1.4)
Not specified	17 (24.6)
Creator gender identity	
Nonbinary	29 (42.0)
Cisgender woman	16 (23.2)
Trans masculine	14 (20.3)
Transgender man	9 (13.0)
Transgender woman	6 (8.7)
Trans feminine	1 (1.4)
Unknown	1 (1.4)
N/A^b^	3 (4.3)
Creator sexual orientation	
Bisexual	10 (14.5)
Queer	10 (14.5)
Lesbian	1 (1.4)
Gay	1 (1.4)
Pansexual	1 (1.4)
Unknown	43 (62.3)
N/A	3 (4.3)
Health care professional	
Yes	17 (24.6)
No or unsure	52 (75.4)
GAHT user	
Yes	49 (71.0)
No or unsure	20 (28.9)
Creator affiliation	
None	65 (94.2)
Health care organization	3 (4.3)
Religious organization	1 (1.4)

a GAHT: gender-affirming hormone therapy.

b N/A: not applicable.

Common themes that appeared in videos are described in Table 3. Self-identified health care workers created 50% of videos about estrogen and antiandrogen regimens, 46% of videos about GAHT access and legality, and 33% of videos about physical changes on testosterone. They created relatively smaller proportions of videos about testosterone regimens (24%), combating misinformation or social stigma around GAHT (11%), and physical changes due to testosterone (10%). None posted antitrans rhetoric.

**Table 3.** Content themes and subthemes appearing in the 69 TikTok videos analyzed.

Content themes and subthemes	n (%), N=69
Physical changes on testosterone	29 (42.0)
Voice changes	20 (29.0)
Facial or body hair growth	11 (15.9)
Clitoral growth	7 (10.1)
Skin changes (acne or oiliness)	6 (8.7)
Body odor changes	3 (4.3)
Body composition changes	6 (8.7)
Physical changes on estrogen	2 (2.9)
Breast development	4 (2.9)
Pre-post therapy photo reveal	17 (24.6)
Testosterone regimens	13 (18.8)
Medication formulations and routes of administration	2 (2.9)
Medication safety or monitoring	2 (2.9)
Concomitant use of estrogen-containing medications	6 (8.7)
Estrogen and antiandrogen regimens	4 (5.8)
Medication formulations and routes of administration	3 (4.3)
Medication safety or monitoring	13 (18.8)
GAHT^a^ access and legality	6 (8.7)
Reviews new state guidelines or policy proposals	4 (5.8)
Reviews the process of obtaining medical clearance or prescription	3 (4.3)
Promotes a health care practice offering GAHT	1 (1.4)
Solicits advice on obtaining a prescription	1 (1.4)
Offers resources for funding GAHT	9 (13.0)
Combating misinformation and social stigma about GAHT	6 (8.7)
Validates TGD^b^ identities	5 (7.2)
Emphasizes mental health benefits of access to GAHT	2 (2.9)
Normalizes the use of GAHT	29 (42.0)

a GAHT: gender-affirming hormone therapy.

b TGD: transgender and gender diverse.

Regarding engagement, videos had a total of 446,318 views, 43,743 likes, 1184 comments, and 438 shares. The sums, medians, and IQRs of engagement measures across each video type are described in Table 4. The top three most viewed videos each discussed physical changes on testosterone therapy and accounted for 46% of total views, 55 of total likes, 32% of total comments, and 56% of total shares. The single most-viewed video (104,800 views) depicted a nurse practitioner discussing labial changes due to testosterone with the aid of a plastic anatomic model.

**Table 4.** Number of views, likes, comments, shares, and creator fans for the 69 TikTok videos analyzed.

Engagement statistics by video type	All, n=69	Personal experience, n=45	Health education, n=15	Politics, n=4	Creator opinion, n=4	Humor, n=1
View count						
Total	440,711	272,602	138,843	18,165	9808	1293
Median (IQR)	847 (283‐2649)	843 (313‐2600)	423 (276‐4315)	1451 (235‐5758)	2122 (1148‐3424)	N/A^a^
Like count						
Total	43,743	26,431	12,281	3671	1051	309
Median (IQR)	77 (21‐248)	77 (28‐222)	25 (11‐242)	149 (19‐1048)	185 (106‐341)	N/A
Comment count						
Total	1169	585	246	226	96	16
Median (IQR)	6 (1-16)	6 (2-13)	2 (0‐9)	34 (0‐91)	23 (19‐28)	N/A
Share count						
Total	437	198	101	118	20	0
Median (IQR)	0 (0‐3)	0 (0‐1)	0 (0‐6)	8 (0‐37)	5 (2-9)	N/A
Creator fans						
Total	488,055	185,202	129,321	162,177	10,857	498
Median (IQR)	1871 (593‐3448)	1871 (759‐3047)	189 (39‐16,648)	3429 (2257‐41,717)	2269 (1924‐4640)	N/A

a N/A: not applicable.

### Quality Rating of the Content

Health education videos averaged 88.3% (SD 11.1%; median 85.7%) on PEMAT-A/V understandability items, and 60.0% (SD 45.8%; median 100%) on actionability items. Together, the weighted mean (SD) PEMAT-A/V score for all items was 81.7% (18.7%). Videos averaged 74.5% (SD 18.4%) in total on the CRAAP test, with mean (SD) component scores as follows: currency, 58.7% (19.2%); relevance, 93.3% (SD 20.0%); authority, 82.7% (SD 35.3%); accuracy, 61.0% (SD +29.3%); and purpose 81.7% (SD 14.1%).

## Discussion

### Principal Findings and Comparison With Previous Works

This study characterized the creators and content of 69 TikTok videos related to GAHT. The most common videos were those made by TGD content creators who shared personal experiences on GAHT. For example, many videos were part of weekly or monthly series in which the creator reviewed the physical effects of their medications or applied or injected medication on camera while providing updates to their followers. Attitudes among users were overwhelmingly positive, with a few instances of ambivalence or mixed attitudes reflecting a preference for one mode of GAHT administration over another.

The large proportion of personal experience videos, which also had high engagement from viewers, reflects the longstanding popularity of TGD video blogs (vlogs) across other social media sites, including Reddit and YouTube [26-28]. As in the current study, prior work has noted an increased frequency of videos created by testosterone users relative to estrogen/anti-androgen users, which may be partially due to the shorter onset time of visible bodily changes with testosterone use [29,30]. Prior YouTube-based ethnographic research suggests these vlogs simultaneously function as opportunities for creators to reflect on and visualize their own gender affirmation journeys, as well as digital diaries to share their narratives with others and engage in broader dialogue [28]. The prevalence of and engagement with personal experience videos in this dataset suggest that TikTok provides a similar space for TGD GAHT users to continually reaffirm their personal identities and engage with the community through digital narrative-sharing.

Our findings suggest that TikTok also functions as a platform for digital activism and resistance. Nearly a third of videos dealt with issues related to GAHT access, newly imposed restrictions on GAHT use, and disinformation about GAHT in the media. In these videos, creators reviewed the process of obtaining a prescription, described sources of funding, or detailed how users could continue to access GAHT despite restrictive legislation passed in Florida and Utah during the timeframe studied. Many used TikTok’s duet function to respond directly to disinformation circulating in the media and among politicians. These videos represent a timely means of intracommunity resistance against restrictive legislation by providing GAHT users with steps to continue GAHT. As legislatures in the United States and abroad attempt to ban and restrict access to care, social media platforms including TikTok may play an increasingly important role in community organizing and harm reduction.

Our results suggest that TikTok provides space for dialogue between GAHT users and health care workers, which may help reduce medical mistrust and facilitate the safe administration of GAHT. Content made by health care workers constituted nearly a quarter of all videos and was created with the intention of sharing health information relevant to GAHT users. These videos showed high levels of engagement, and often directly addressed viewers’ comments or private messages. The ability for health care providers to respond directly to GAHT users’ questions about hormones supports its potential as a space for effective digital knowledge mobilization, as prior work suggests [2]. Furthermore, health care providers also benefit from this interaction by gaining a deeper understanding of common questions and concerns among TGD people interested in GAHT.

Our analysis of health education videos suggests that information contained in educational videos is of high understandability, low actionability, and moderate reliability. An average PEMAT understandability score of 88.3% across educational videos suggests a high level of accessibility to viewers. The lower average actionability score of 60.0% may reflect the fact that the goal of many health education videos was to explain a particular phenomenon (eg, the mechanisms behind expected physical changes due to testosterone) rather than to guide patients’ decisions about treatment.

High scores on the relevance, authority, and purpose components of the CRAAP test (>80%) suggest that educational information regarding GAHT is well-suited to the needs of the intended audience, created by reliable sources, and shared with the purpose of informing viewers about their health. The lower scores on the accuracy and currency components of the scale reflect the finding that while many content creators used their professional experience to support their claims, few cited evidence-based guidelines or provided viewers with further reading or updates as new information emerged. While the content analyzed was overall understandable, it did not reliably contain the level or depth of detail present in evidence-based clinical practice guidelines. It may therefore be important for providers to work with patients to contextualize information about GAHT found on the platform.

### Clinical Implications

Our findings have several clinical implications. Providers should be aware that patients may use TikTok as a source of health knowledge, and that this information varies in depth and accuracy. GAHT prescribers may consider incorporating routine screening questions about patients’ consumption of medication-related social media content and using these to either augment, contextualize, or correct information found online. As much as GAHT users may use TikTok as a space to seek information from health care providers, it can also better inform providers about TGD patients’ needs and priorities. These needs are apparent where videos made by providers and users diverge thematically. For instance, providers created a high proportion of videos surrounding drug safety and monitoring, whereas GAHT users focused more on desired medication effects, suggesting a potential need for comprehensive counseling on expected and adverse effects.

Furthermore, videos created by providers also differed from user-created videos in that few of them directly addressed a nonbinary audience, despite nonbinary content creators being the most well-represented demographic in the videos analyzed, suggesting that there may be a lack of awareness surrounding GAHT-related needs of nonbinary people. In fact, only one health education video created by a provider explicitly addressed a nonbinary audience, and nearly a quarter used language that reinforced a binary gender paradigm (eg, “this video is for anyone transitioning male to female”). Thus, TikTok may also offer cisgender providers an opportunity to better understand the unique needs of diverse groups seeking out GAHT, which may allow for a more patient-centered and culturally responsive approach to counseling.

### Limitations

There are several limitations to this study. First, the content analyzed is limited to what appears under the specific search terms #genderaffirminghormonetherapy and #genderaffirming hormones. While there is likely broader discourse on TikTok surrounding this topic, broadening the search terms—for example, to #hormonetherapy or #testosterone—would have yielded results that are not specific or relevant to TGD communities. While the videos analyzed were created over a 20-month period, they represent a single snapshot in time within the rapidly changing landscape of social media.

We were unable to compare quantitative statistics between different video types due to the small sample size for some video types and the non-normal distribution of engagement statistics with videos that were outliers with regard to viewership. Future studies should conduct rigorous statistical comparisons on video metrics in a larger sample size.

Additionally, TikTok has been criticized for its tendency toward “collaborative filtering,” a method of predicting users’ interests based on their previous views and activity in the app. By using physiognomic data, some argue that TikTok is more likely to recommend creators who look like the platform’s white and able-bodied top influencers, and less likely to recommend creators who belong to underrepresented minority groups, which can also be referred to as “shadow banning” [31-33]. In this context, it is important to consider that some perspectives may be systematically privileged over others.

Similarly, the collected data may be vulnerable to bias towards more positive experiences. Users may be more willing to share positive experiences given the nature of the community formed under these hashtags; those with negative experiences may be less willing to share their experiences. However, we attempted to mitigate bias in content selection by using a web scraper rather than a TikTok account for data collection.

Our analysis involved collecting self-reported demographic characteristics from public profiles, which can be falsely reported for a variety of reasons, including stigma or safety concerns. Thus, it is possible that the number of TGD content creators and GAHT users within the dataset was underreported. Moreover, we chose to limit our focus to video content to analyze the dialogue between creators and viewers, though future research may also include the comment sections of such videos.

Finally, while an abundance of clinical evidence supports the efficacy of GAHT, there remains debate nationally and internationally on certain aspects of GAHT. Videos were rated based on the contemporary understanding of GAHT, with the authorship team comparing information presented in videos to the majority consensus in the academic field; however, we acknowledge that our ratings are limited by a lack of consensus in the clinical community on certain topics related to GAHT.

### Conclusions

This study evaluated the discourse around GAHT on TikTok to better understand the extent to which it is being used as a tool for building community and disseminating health knowledge. Overall, our results suggest that TikTok allows GAHT users to document their experiences, connect with other community members, and advocate for GAHT as legislation restricts access to treatment. TikTok also provides a space for direct user-provider dialogue, whereby users can have questions answered by health professionals with a high level of information understandability. Health professionals should be aware that patients may use TikTok as a source of information and should be ready to explore these sources of knowledge with patients, as they vary in terms of currency and quality of supporting evidence. Health care workers may utilize social media platforms such as TikTok as an opportunity for bidirectional learning and health knowledge dissemination between clinicians and GAHT users.

## Abbreviations

CRAAP: Currency, Relevance, Authority, Accuracy, and Purpose
GAHT: gender-affirming hormone therapy
LGBTQIA+: lesbian, gay, bisexual, transgender, queer, intersex, and all asexually and gender diverse
PEMAT-A/V: Patient Education Materials Assessment Tool for Audiovisual Materials
TGD: transgender and gender diverse
